# Clinical diagnosis of patients subjected to surgical lung biopsy with a probable usual interstitial pneumonia pattern on high-resolution computed tomography

**DOI:** 10.1186/s12890-020-01339-9

**Published:** 2020-11-16

**Authors:** Regina Celia Carlos Tibana, Maria Raquel Soares, Karin Mueller Storrer, Gustavo de Souza Portes Meirelles, Katia Hidemi Nishiyama, Israel Missrie, Ester Nei Aparecida Martins Coletta, Rimarcs Gomes Ferreira, Carlos Alberto de Castro Pereira

**Affiliations:** 1 Pulmonary Department, Federal University of Sao Paulo, R. Botucatu, 740 - Vila Clementino, São Paulo, SP 04023-062 Brazil; 2 Imaging Department, Fleury Group, Sao Paulo, Brazil; 3 Radiology Department, Federal University of Sao Paulo, Sao Paulo, Brazil; 4 Pathology Department, Federal University of Sao Paulo, Sao Paulo, Brazil

**Keywords:** Idiopathic pulmonary fibrosis, Hypersensitivity pneumonia, Usual interstitial pneumonia, Interstitial lung disease, Surgical lung biopsy, High-resolution computed tomography

## Abstract

**Background:**

Usual interstitial pneumonia can present with a probable pattern on high-resolution computed tomography (HRCT), but the probability of identifying usual interstitial pneumonia by surgical lung biopsy in such cases remains controversial. We aimed to determine the final clinical diagnosis in patients with a probable usual interstitial pneumonia pattern on HRCT who were subjected to surgical lung biopsy.

**Methods:**

HRCT images were assessed and categorized by three radiologists, and tissue slides were evaluated by two pathologists, all of whom were blinded to the clinical findings. The final clinical diagnosis was accomplished via a multidisciplinary discussion. Patients with a single layer of honeycombing located outside of the lower lobes on HRCT were not excluded.

**Results:**

A total of 50 patients were evaluated. The most common final clinical diagnosis was fibrotic hypersensitivity pneumonitis (38.0%) followed by idiopathic pulmonary fibrosis (24.0%), interstitial lung disease ascribed to gastroesophageal reflux disease (12.0%) and familial interstitial lung disease (10.0%). In the group without environmental exposure (*n* = 22), 10 patients had a final clinical diagnosis of idiopathic pulmonary fibrosis (45.5%). Irrespective of the final clinical diagnosis, by multivariate Cox analysis, patients with honeycombing, dyspnoea and fibroblastic foci on surgical lung biopsy had a high risk of death.

**Conclusions:**

The most common disease associated with a probable usual interstitial pneumonia pattern on HRCT is fibrotic hypersensitivity pneumonitis followed by idiopathic pulmonary fibrosis and interstitial lung disease ascribed to gastroesophageal reflux disease. In patients without environmental exposure, the frequencies of usual interstitial pneumonia and a final clinical diagnosis of idiopathic pulmonary fibrosis are not sufficiently high to obviate the indications for surgical lung biopsy.

**Supplementary Information:**

The online version contains supplementary material available at 10.1186/s12890-020-01339-9.

## Background

Idiopathic pulmonary fibrosis (IPF) is a fibrosing interstitial lung disease (ILD) of unknown aetiology defined by the presence of usual interstitial pneumonia (UIP) pattern on surgical lung biopsy (SLB) [1]. On high-resolution computed tomography (HRCT), the UIP pattern is characterized by predominant basal reticular abnormalities with honeycombing cysts and the absence of inconsistent features and is considered sufficient for diagnosis in the proper clinical context [1]. However, many patients with IPF do not have a UIP pattern on HRCT. Patients with the same findings of the UIP pattern except for honeycombing were classified by the American Thoracic Society/European Respiratory Society/Japanese Respiratory Society/Latin American Thoracic Association (ATS/ERS/JTS/ALAT) in 2011 as having possible UIP [1].

Some studies found a high positive predictive value of a possible UIP pattern on HRCT for the diagnosis of UIP on lung biopsy [2–6]. However, these studies included patients with a high prevalence of UIP, which introduced a selection bias and inflated the diagnosis of IPF. A study of individuals with possible UIP patterns on HRCT showed that the positive predictive value is highly dependent on the underlying prevalence [5]. In that study, the second most common diagnosis was hypersensitivity pneumonitis (HP). In Brazil, HP is a common cause of ILD [7].

More recently, the Fleischner Society and ATS/ERS/JTS/ALAT guidelines suggested four categories for classifying HRCT patterns of UIP [8, 9]. In the absence of honeycombing and in the appropriate clinical context, a reticular pattern with lower lobe predominance and traction bronchiectasis should not be considered a possible UIP pattern but rather a probable UIP pattern. According to the Fleischner Society, SLB can be avoided in such cases, but the ATS/ERS/JRS/ALAT made a conditional recommendation for SLB in these cases [8, 9].

The objective of the present study was to evaluate the frequency of IPF and other ILDs in patients with a probable UIP pattern on HRCT.

## Methods

This study was approved by the Ethics and Research Committee of the Federal University of Sao Paulo (register number 2.180.594). Informed consent was not required as the data were collected retrospectively and anonymously analysed.

### Study population

Patients were selected retrospectively from ILD reference centres in Brazil. The period of the study was from 2002 to 2018. For inclusion in the study, HRCT, histological samples, and clinical data were available for central review and a multidisciplinary discussion (MDD). All HRCT images were obtained within 1 year of the biopsy. Consecutive patients were evaluated until 50 filled the criteria for analysis.

### Inclusion criteria

The inclusion criterion was the presence of a probable UIP pattern on HRCT according ATS/ERS/JRS/ALAT 2018 guidelines [9].

### Exclusion criteria

Patients with any one of the following HRCT UIP patterns were excluded: UIP or indeterminate or alternative diagnosis of UIP, including predominant bronchovascular abnormalities [9]; furthermore, those with inadequate HRCT image quality; emphysema with extension greater than 10% on HRCT; diagnosis of connective tissue disease prior to SLB; and a probable UIP pattern on HRCT with exposure to HP and lymphocytosis (> 20%) on bronchoalveolar lavage were also excluded. Patients with a single layer of honeycombing cysts located outside of the lower lobes on HRCT were not excluded.

### Clinical data

Demographic data were recorded. Dyspnoea was scored as absent, major, moderate, small effort or at rest, according Mahler’s scale for magnitude of task [10]. Gastroesophageal reflux disease (GERD) symptoms investigated included heartburn, regurgitation and dyspepsia. GERD was evaluated by endoscopy, oesophageal manometry and pH monitoring [11, 12].

Familial ILD was defined as the diagnosis of an ILD in two or more relatives who shared common ancestry [13].

The predicted values for forced vital capacity (FVC) were those derived from the Brazilian population [14].

### HRCT evaluation

All HRCT scans were reviewed through a protocol by three thoracic radiologists (GSPM, IM and KN) with experience in ILD (18, 22 and 8 years, respectively) without knowledge of the clinical data and final clinical diagnosis. The presence of a probable UIP pattern in each patient was established by a consensus. Patients in whom no consensus was reached among radiologists for probable UIP were excluded.

### Histologic evaluation

All samples were reviewed independent of each other by two pathologists (RGF and ENAMC) with experience in ILD (35 and 27 years, respectively).

The histological pattern of UIP was characterized according to the criteria proposed for definitive and probable UIP in 2011 by the ATS/ERS/JRS/ALAT [1].

Bronchiolocentric fibrosis (BF) was defined by predominant involvement centred on the airways associated with inflammation or peribronchiolar metaplasia [15]. The presence of giant cells and granuloma, fibroblastic foci (FF) and microscopic honeycombing was noted. The number of FF was considered relevant if greater than occasional [16].

Histological findings suggestive of autoimmune disease were characterized by the presence of lymphocytic pleuritis, bronchiolitis, vascular sclerosis, several lymphoid follicles, and intense lymphoplasmacytic infiltrate [17].

Unclassifiable ILD was defined as the presence of overlapping patterns found in a single lobe or multiple lobes or when it was not possible to include the patient in any of the categories proposed for the classification of interstitial pneumonias [18].

### Final clinical diagnosis

The patients were reassessed, and the final clinical diagnosis was established by an MDD with the same pathologists and three pulmonologists experienced in ILD (RCCT, MRS, and CACP). IPF was defined as definitive or probable histological patterns of UIP in the absence of other potential aetiologies [1]. In the presence of environmental exposure, the diagnosis was still defined as IPF in the presence of a UIP pattern in more than one lobe, without any other histological findings suggestive of HP. [19] Fibrotic HP (FHP) was defined as the presence of environmental exposure before the onset of symptoms and by the presence of one of the following histological findings: 1) BF and/or lymphomononuclear infiltrate, bronchiolar poorly defined nonnecrotizing granulomas, and/or giant cells and bronchiolitis or 2) BF with or without giant cells or granulomas in the absence of GERD [20].

For the diagnosis of ILD ascribed to GERD, the patients had to fulfil the following criteria [15] in the presence or absence of symptoms of GERD: histological patterns of BF in the absence of environmental exposure to HP and GERD confirmed through one or more of the following: oesophagitis on upper gastrointestinal endoscopy or abnormal oesophageal pH monitoring characterized by a DeMeester score greater than 14.7 or proximal reflux characterized by 1% or more of the total time with a pH less than 4 at the proximal sensor [11, 12].

The clinical diagnosis of familial ILD was maintained irrespective of the histopathologic findings [21]. Interstitial pneumonia with autoimmune features was characterized as suggested by the ATS/ERS task force [22].

### Statistical analysis

Continuous data are expressed as the mean and standard deviation or as the median and interquartile range. Categorical variables are described as absolute numbers and percentages. The values for the most common final clinical diagnosis and histologic patterns are expressed as the mean percentage and 95% confidence interval of proportions. The comparison between categorical variables was performed using Fisher’s exact chi-square test.

The test characteristics of the probable UIP pattern (sensitivity, specificity and positive predictive value) for histopathological UIP were calculated using the prevalence of IPF observed in Brazil (10%) and in other countries with the highest prevalence [7, 23, 24].

Survival time was calculated from the date of biopsy to death, lung transplantation (*n* = 1) or loss to follow-up. The survival status was obtained from telephone interviews and/or medical records. The follow-up time was censored on April 30, 2019. All-cause mortality was evaluated.

Univariate Cox analysis was performed to select variables related to survival, and those with *p* values < 0.20 were entered into a multivariate forward Wald Cox model to select variables predictive of survival. Kaplan-Meier curves were generated to calculate the median survival time and to compare survival between patients with and without the variables of interest.

A *p* value < 0.05 was considered statistically significant.

Statistical analysis was performed using SPSS software version 21 (IBM, Armonk, NY, USA).

## Results

One hundred seventy-seven patients with HRCT images available for re-reading and an SLB sample available for review were selected. After review, 127 patients were excluded (Supplementary Fig. S1). Ultimately, 50 patients were included. The general characteristics are described in Table 1.

**Table 1:** General features of 50 patients with a probable UIP pattern on HRCT

Characteristic	
Age in years, $\overline{x}$ ± SD	64.0 ± 7.4
Sex, male, n (%)	32 (64.0)
Duration of symptoms in months, median (Q1-Q3) (*n* = 47)	15.0 (6.8–36.0)
Smoking history, n (%)	26 (52.0)
Environmental exposure, n (%)	28 (56.0)
Family history, n (%)	5 (10.0)
Dyspnoea, any, n (%)	41 (82.0)
Cough, yes, n (%)	32 (64.0)
Symptoms of GERD, n (%)	22 (44.0)
GERD confirmed, n (%)	16 (32.0)
Velcro crackles, n (%)	34 (68.0)
FVC% predicted, $\overline{x}$ ± SD	80.9 ± 16.8
FEV_1_% predicted, $\overline{x}$ ± SD	85.6 ± 17.9
FEV_1_/FVC, $\overline{x}$ ± SD	0.84 ± 0.08
SpO_2_ rest % (*n* = 44), $\overline{x}$ ± SD	95.0 ± 1.9
Honeycombing on HRCT^a^, n (%)	8 (16.0)

^a^Patients with a single layer of honeycombing cysts located outside of the lower lobes

*HRCT* High-resolution computed tomography, *GERD* Gastroesophageal reflux disease, *FVC* Forced vital capacity, *FEV*_*1*_ Forced expiratory volume in the first second, *SpO*_*2*_ Peripheral oxygen saturation

Environmental exposure to HP was reported in 28 patients (56%): mould (*n* = 10), birds/feathers (*n* = 11), mould and birds (*n* = 4), and wood dust (*n* = 3).

GERD symptoms were also common and were reported in 22 (44%) patients.

Honeycombing located outside of the lower lobes was recorded in eight patients (16.0%).

The histological patterns are described in Table 2. BF was the most commonly observed histological pattern in 26 (52.0%) patients. The classical histological triad of HP was found in seven (14%) patients, and BF with giant cells and/or granulomas was found in five (10%) patients, two of whom had histological findings of FF and/or microscopic honeycombing. Bridging fibrosis were observed in four patients with BF. The second most prevalent pattern was UIP (26%). Unclassifiable ILD and interstitial pneumonia with autoimmune features were found in two (4%) patients each.

**Table 2:** Histological patterns of 50 patients with a probable UIP pattern on HRCT

Histological pattern	n (%)
Bronchiolocentric fibrosis	26 (52.0)
With fibroblastic foci and/or microscopic honeycombing	14 (28.0)
With giant cell and/or granulomas	3 (6.0)
With fibroblastic foci and/or microscopic honeycombing plus granulomas and/or giant cells	2 (4.0)
Without other findings	7 (14.0)
Usual interstitial pneumonia	13 (26.0)
Classical histological triad of hypersensitivity pneumonitis	7 (14.0)
Unclassifiable interstitial lung disease	2 (4.0)
Interstitial pneumonia with autoimmune features	2 (4.0)

The final clinical diagnoses are described in Table 3. The most frequent diagnosis was FHP followed by IPF and ILD ascribed to GERD. After excluding patients with environmental exposure (*n* = 22), 10 (45%) patients were diagnosed with IPF. One patient without apparent exposure but with typical findings on SLB received a final clinical diagnosis of FHP.

**Table 3:** Final clinical diagnoses after a multidisciplinary discussion

Final clinical diagnosis	n (%)
Fibrotic hypersensitivity pneumonitis	19 (38.0)
Idiopathic pulmonary fibrosis	12 (24.0)
Interstitial lung disease ascribed to GERD	6 (12.0)
Familial interstitial lung disease	5 (10.0)
Unclassifiable interstitial lung disease	2 (4.0)
Fibrotic hypersensitivity pneumonia and/or interstitial lung disease ascribed to GERD	2 (4.0)
Interstitial pneumonia with autoimmune features	2 (4.0)
Idiopathic bronchiolocentric fibrosis	2 (4.0)

The final diagnosis established among the 28 patients with environmental exposure were: 18 FHP; three patients with familial ILD; two unclassifiable ILD; two IPF; one IPAF; in two patients, the final clinical diagnosis was FHP and/or ILD ascribed to GERD due to the presence of BF on histology, relevant environmental exposure and confirmed GERD.

In two patients with histological findings of BF, no cause was apparent (idiopathic BF).

After the MDD, the two patients with histological patterns of unclassifiable ILD remained without a final clinical diagnosis.

In five patients, there was a familial history of ILD, which was the final clinical diagnosis. These patients presented with the following histological patterns: BF (*n* = 3), UIP (*n* = 1) and classical histological triad of HP (*n =* 1).

Groups of patients with FHP, IPF and the other diagnoses were compared. As expected, the presence of environmental exposure was different between the groups: 20 cases (95.2%) in the FHP group, two cases (16.7%) in the IPF group and 11 cases (64.7%) in the other diagnoses group (χ^2^ = 21.8; *p* < 0.001). Confirmed GERD was more frequent in the group with other diagnoses: 11 cases (64.7%) compared to three cases (15%) in the FHP group and two cases (16.7%) in the IPF group (χ^2^ = 13.7; *p* = 0.002). The presence of Velcro crackles, forced vital capacity (FVC%), and peripheral oxygen saturation at rest and at the end of exercise were similar in the three groups (Supplementary Table S1).

In the present study, no patient died in the first month after SLB. The median general survival time was 72.0 (95% CI = 57.5–96.5) months. According to Kaplan-Meier curves, the median survival time was similar when patients with IPF, FHP and other diagnoses were compared (Supplementary Fig. S2).

Honeycombing outside of the lower lobes was present in four patients with FHP, one with IPF, and three with other diagnoses. Fibroblastic foci was observed in all 12 patients with IPF, eight patients with FHP and 12 patients with others diagnosis: five familial ILD; three ILD ascribed to GERD; two idiopathic BF; one IPAF and one case that was classified as HP plus ILD ascribed to GERD. In the univariate Cox analysis, age, dyspnoea score, FVC%, FF and honeycombing on HRCT were selected. In the multivariate Cox analysis, honeycombing outside of the lower lobes, dyspnoea score and FF on SLB remained significant (Table 4). When the FVC% was entered in the model instead of dyspnoea, it became significant, with lower values associated with poorer survival (*p* = 0.01).

**Table 4:** Multivariate analyses for survival^a^

Factor	HR	95% CI	*p*-value
Honeycombing on HRCT^b^	11.9	2.9–55.0	0.001
Dyspnoea score	2.9	1.6–6.0	0.004
Fibroblastic foci on SLB	6.2	1.6–24.3	0.009

^a^Model containing age, dyspnoea score, % predicted forced vital capacity, fibroblastic foci on SLB, and honeycombing on HRCT

^b^A single layer of honeycombing cysts located outside of the lower lobes

*HRCT* High-resolution chest tomography, *SLB* Surgical lung biopsy

Antifibrotic agents were prescribed for 13 patients, including seven with IPF.

After excluding environmental exposure (*n* = 28) as a possible cause of the number of FHP cases diagnosed, a probable UIP pattern on HRCT demonstrated a sensitivity and specificity of 83.3% (95% CI 51.6 to 97.9%) and 68.4% (95% CI 51.3 to 82.5%), respectively. The positive predictive value was 22.7% (95% CI 14.7 to 33.3%) when considering the prevalence of IPF as 10 and 63.7% (95% CI 50.8 to 74.9%) when considering the prevalence of IPF as 40%.

## Discussion

In the present study, FHP was the most frequent cause of a probable UIP pattern on HRCT. Considering only the patients without a history of environmental exposure, IPF was observed in less than 50% of the sample, with several ILDs diagnosed in the remaining subjects.

In 2011, the ATS proposed a tomographic classification for UIP in three categories [1]. In 2017, the Fleischner Society suggested splitting the possible group from the ATS 2011 guidelines into the probable and indeterminate groups [8]. Probable UIP was characterized by the presence of a reticular pattern with peripheral traction bronchiectasis or bronchiolectasis in the absence of honeycombing. A diagnosis of IPF could confidently be made in a patient with a typical clinical context of IPF, with an HRCT pattern of probable UIP [8]. This statement was based on two papers: one had a recognized selection bias [3], and the other included patients with an undefined location of honeycombing on HRCT as a probable UIP pattern [4].

Similar to the Fleischner Society, in 2018, the ATS/ERS/JRS/ALAT suggested a pattern of “probable UIP”, but the panel emphasized that the decision to perform SLB should be made in the context of an MDD by experienced clinicians [9].

Criteria for the diagnosis of IPF have been discussed for a long time. Criteria for the diagnosis of FHP have been suggested more recently, but many differences persist [25–27]. Relevant environmental exposure, suggestive HRCT findings, and increased lymphocytes in bronchoalveolar lavage are important for diagnosis but are not helpful in many cases. Histopathologic findings can be typical, but these are absent in many cases of FHP.

In Brazil, HP is more common than IPF as a cause of ILD (24 vs 10%), as shown in a recent multicentre study [7]. However, 53% of all patients with ILD had potential exposure to HP, raising the question of whether other diagnoses can be excluded simply by the presence of environmental antigens. In the present study, only one patient without apparent exposure had a diagnosis of FHP established by typical findings on biopsy. According to the 2020 Guideline for diagnosis of HP, this case would be classified as “high confidence diagnosis” (HRCT with indetermined findings plus typical histopathologic findings) [28].

In contrast, in many studies, the causal agent for HP has not been identified [26]. Indeed, the prevalence of HP can be underestimated. In a study from Spain, almost half of the patients diagnosed with IPF based on the 2011 criteria were subsequently diagnosed with FHP [29].

HP can present with several histopathologic patterns [19]. In FHP, a typical pattern is characterized by chronic fibrosing pneumonia, airway-centred fibrosis and poorly formed nonnecrotizing granulomas. Fibrosing interstitial pneumonia is characterized by architectural distortion; FF and subpleural honeycombing can be present. In airway-centred fibrosis or BF, there is also extensive peribronchiolar metaplasia and, in many cases, bridging fibrosis. In FHP, the presence of granulomas and giant cells is uncommon. Another possible presentation for FHP is a pattern of nonspecific interstitial pneumonia.

In the present study, six patients were diagnosed with ILD ascribed to GERD. In these patients, the histological pattern was BF, there was no environmental exposure to HP, and GERD was substantiated. In our opinion, the role of GERD as an aetiological factor of fibrosing ILD, not UIP, has been neglected [15].

In two patients in the present series with BF, FHP and ILD ascribed to GERD were possible causes. In our opinion, these patients cannot be discriminated by biopsy. In the other two patients, a possible cause for BF was not determined.

Patients with nonspecific interstitial pneumonia were not observed in the present series, probably due to a high frequency of findings inconsistent with UIP on HRCT.

In the present study, five patients had familial ILD. On SLB, three displayed BF with FF and/or microscopic honeycombing, one had a typical PH pattern, and one had an isolated UIP pattern. In a study of 30 patients with familial ILD, diagnostic features of UIP were observed in less than 50% of the samples, but FF were observed in 87%, and multifocal BF was observed in 37% [21]. Familial ILD presents in many different ways on HRCT and in lung biopsies [13, 30]. Genetic factors can predispose patients to several ILDs, and diverse pathologic expressions can be found in the same family [13, 31]. The indications for SLB in familial ILD remain controversial [32, 33].

The likelihood of a histopathological UIP pattern in patients with a probable UIP pattern on HRCT remains undefined.

In a recent paper from Japan, the prevalence of a histopathological UIP pattern was 83% (90 of 109) in patients with a probable UIP pattern on HRCT, but the diagnosis of IPF after the MDD was made in only 66% [32]. The median survival time was 72.1 months for patients with the probable UIP pattern, which was very similar to that found in our series. This survival time was longer than that found in patients with IPF, suggesting that a heterogeneous number of diagnoses were included in the group with a probable UIP pattern. In this study, different clinical diagnoses were not the objective and were not explored [34].

In the present study, the degree of dyspnoea, the presence of honeycombing not in the lower lobes irrespective of diagnosis, and the presence of FF on SLB were predictive of poor survival. The presence of honeycombing on HRCT in an ILD other than IPF is a predictor of a poor prognosis, but in most cases, honeycombing is present in the lower lobes [35]. Even with a limited sample, we found that honeycombing outside of the lower lobes was associated with poor survival, irrespective of the final diagnosis.

FF are a major histological feature of UIP in SLB but can be present in other conditions. It has been recognized for a long time that the presence and extent of FF predict poor survival in patients with IPF as well as in those with FHP [16, 20, 36, 37].

In the INPULSIS trial, which evaluated the efficacy and safety of nintedanib in the treatment of IPF, subjects enrolled with the possible UIP pattern and traction bronchiectasis showed similar disease progression and treatment responsiveness to subjects enrolled with the IPF HRCT pattern [38]. This was used as an argument supporting these cases as IPF.

The INBUILD trial was a randomized, double-blind, multicentre, parallel group trial performed in 663 patients with a progressive fibrosing ILD other than IPF [39, 40]. Chronic HP was diagnosed in 173 patients (26%). Participants were randomly assigned to receive 150 mg of nintedanib twice daily or placebo for at least 52 weeks. Nintedanib reduced the rate of ILD progression, as measured by a decline in the FVC, irrespective of the underlying ILD diagnosis. In patients with a UIP-like fibrotic pattern, the adjusted rate of decline in the FVC over the 52-week period was more conspicuous [40].

These studies raise the question of whether the diagnosis of fibrosing ILD truly matters. In FHP, if the antigen is not identified, the prognosis is worse [41]. Otherwise, identification and antigen avoidance, even in FHP, can result in a prolonged survival time [42]. A subset of patients with FHP experience a progressive disease course, even after antigen avoidance, and these patients can be treated with pharmacologic agents, including antifibrotic drugs if necessary.

In patients with fibrotic ILD of indeterminate aetiology, we currently recommend transbronchial lung cryobiopsy before entertaining SLB [43].

Some authors suggest that older age, male sex and smoking could increase the positive predictive value for IPF diagnostics in patients with a possible UIP pattern on HRCT [2]. In the present study, these data were not helpful.

Several limitations to our study should be noted. The sample size was relatively small. This was a retrospective study, and selection bias should be considered. All the patients were reviewed by two expert pathologists in ILD, but the concordance was not evaluated. HRCT during expiration was not performed in all patients; thus, air trapping was not evaluated; however, this finding has a lower predictive value for separating FHP from IPF compared to that for the mosaic pattern [44]. Treatment was not standardized, making it difficult to evaluate its effect on survival.

## Conclusions

Different ILDs are observed in patients with a probable UIP pattern on HRCT. Even in patients without relevant environmental exposure, IPF should not be presumed as the most common diagnosis. After considering the risks, a biopsy approach should be entertained.

## Supplementary Information

**Additional file 1: Fig. S1.** Inclusion criteria diagram. **Fig. S2.** Probable usual interstitial pneumonia pattern on high resolution chest tomography. **Fig. S3.** Fibrotic hypersensitivity pneumonitis. **Fig. S4.** Idiopathic pulmonary fibrosis. **Fig. S5.** Interstitial lung disease ascribed to gastroesophageal reflux disease. **Fig. S6.** Survival in patients with a probable UP pattern on HRCT was analysed according to the main diagnostic groups. **Table S1.** Clinical characteristics, functional characteristics, and HRCT findings of 50 patients with fibrotic chronic hypersensitivity pneumonia, IPF, and other diagnoses and a probable UIP HRCT pattern.

## Data Availability

The datasets supporting the conclusions of this article are included within the article and its additional supplemental files.

## Abbreviations

ATS/ERS/JRS/ALAT: American Thoracic Society/European Respiratory Society/Japanese Respiratory Society/Latin American Thoracic Association
BF: Bronchiolocentric fibrosis
FF: Fibroblastic foci
FHP: Fibrotic hypersensitivity pneumonitis
FVC: Forced vital capacity
GERD: Gastroesophageal reflux disease
HP: Hypersensitivity pneumonitis
HRCT: High-resolution computed tomography
ILD: Interstitial lung disease
IPF: Idiopathic pulmonary fibrosis
MDD: Multidisciplinary discussion
SLB: Surgical lung biopsy
UIP: Usual interstitial pneumonia
95% CI: 95% confidence interval
