# Supplementary material for: Clinical diagnosis of patients subjected to surgical lung biopsy with a probable usual interstitial pneumonia pattern on high-resolution computed tomography
Source: BMC Pulm Med. 2020 Nov 16;20:299. doi: 10.1186/s12890-020-01339-9 (PMC7670778; doi:10.1186/s12890-020-01339-9)
Supplement: Supplementary file 1 — Additional file 1: Fig. S1. Inclusion criteria diagram. Fig. S2. Probable usual interstitial pneumonia pattern on high resolution chest tomography. Fig. S3. Fibrotic hypersensitivity pneumonitis. Fig. S4. Idiopathic pulmonary fibrosis. Fig. S5. Interstitial lung disease ascribed to gastroesophageal reflux disease. Fig. S6. Survival in patients with a probable UP pattern on HRCT was analysed according to the main diagnostic groups. Table S1. Clinical characteristics, functional characteristics, and HRCT findings of 50 patients with fibrotic chronic hypersensitivity pneumonia, IPF, and other diagnoses and a probable UIP HRCT pattern. [file 12890_2020_1339_MOESM1_ESM.docx]

Supplementary items for Clinical diagnosis of patients subjected to surgical lung biopsy with a probable usual interstitial pneumonia pattern on high-resolution computed tomography

Supplementary Figure S1. Inclusion criteria diagram


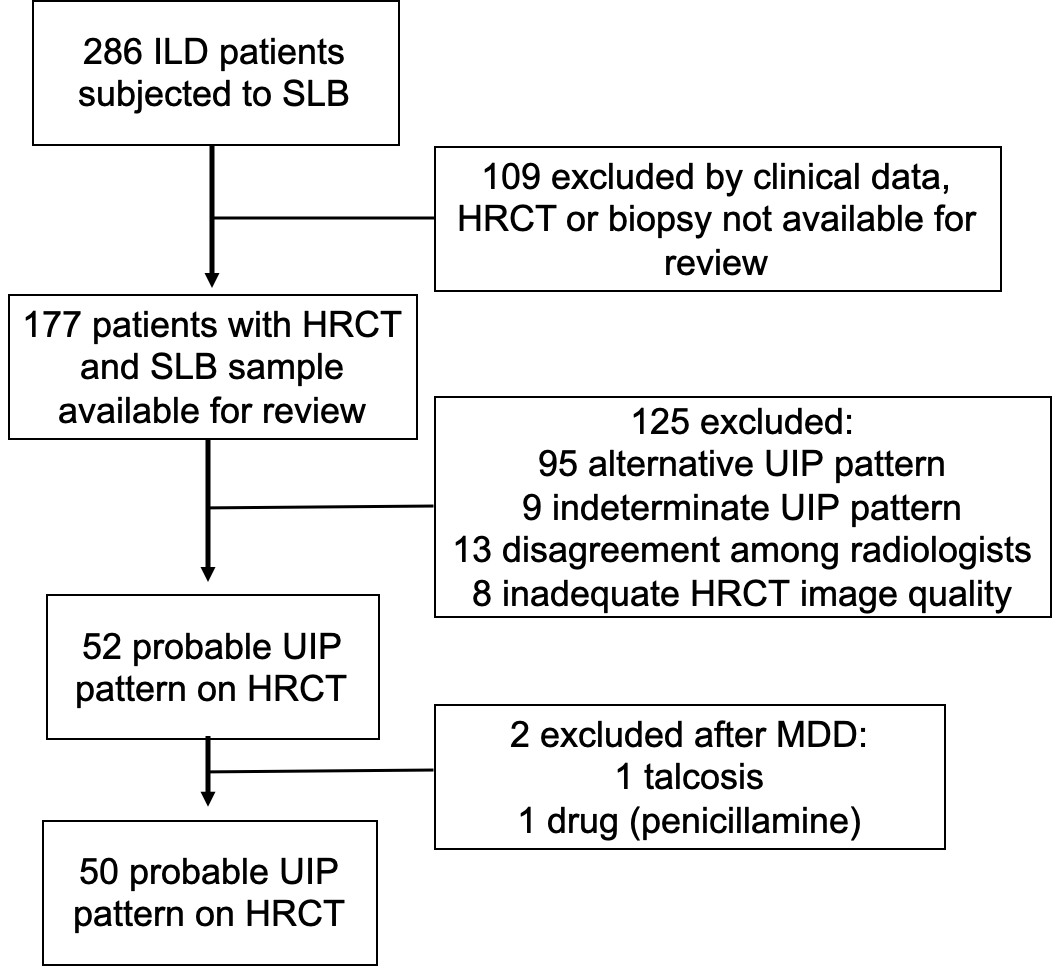


ILD: Interstitial lung disease; SLB: Surgical lung biopsy; HRCT: High resolution computed tomography; UIP: Usual interstitial pneumonia; MDD: Multidisciplinary discussion.

Supplementary Figure S2. Chest high resolution computed tomography (HRCT) in a 60 years-old female patient with probable usual interstitial pneumonia pattern on HRCT and histological bronchiolocentric fibrosis. The final clinical diagnosis was hypersensitivity pneumonitis due to the environmental exposure to mould. Coronal and axial HRCT images show a reticular pattern and traction bronchiolectasis with predominant distribution in the lower lung fields without honeycombing.


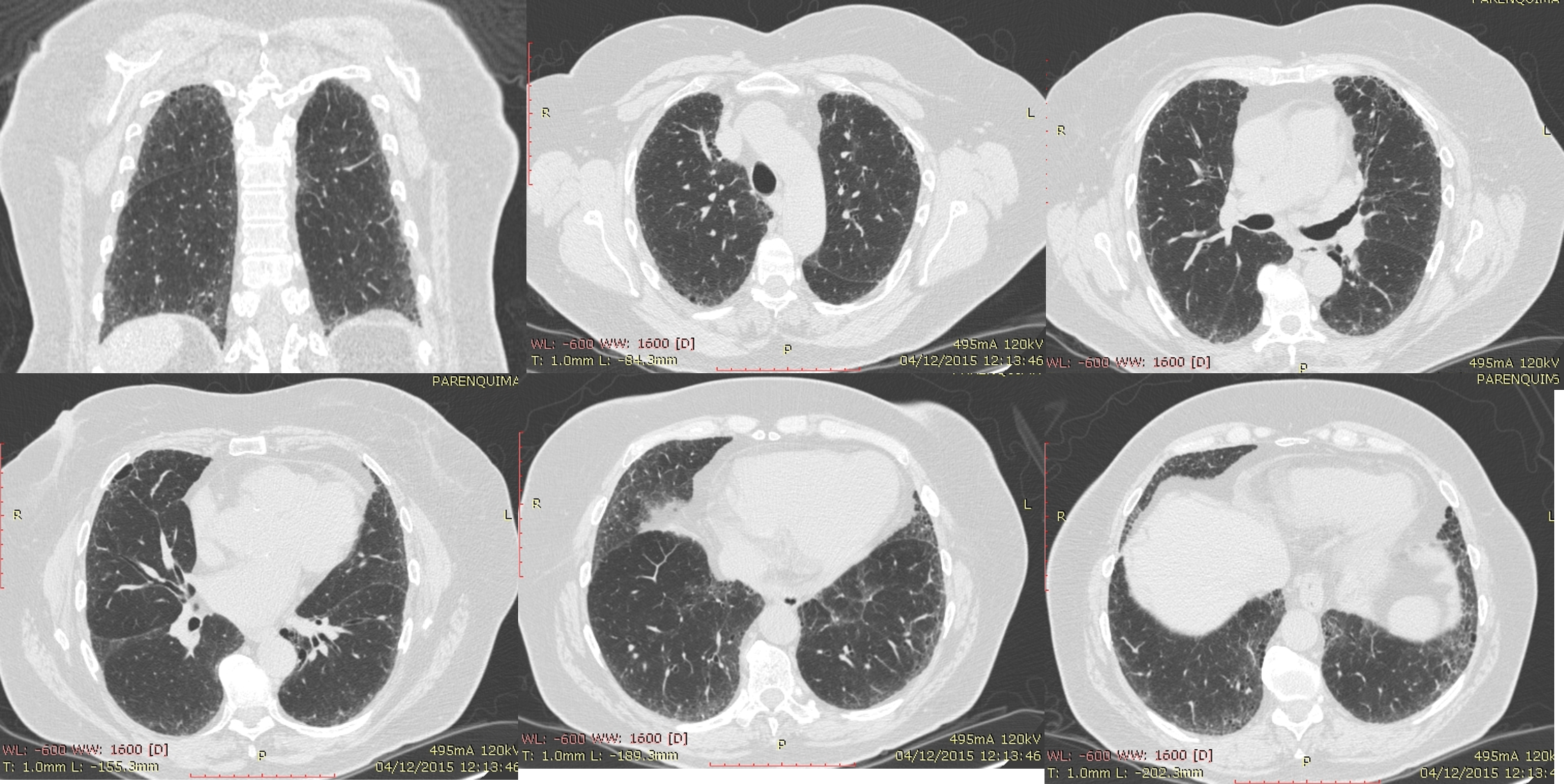


Supplementary Figure S3. Fibrotic hypersensitivity pneumonitis with probable usual interstitial pneumonia pattern on high resolution computed tomography and the classical histological triad of HP. A: Airway-centered fibrosis and peribronchiolar metaplasia (HE, 10x); B: Bronchiolocentric fibrosis (HE, 10x); C: Peribronchiolar giant cells (HE, 20x).


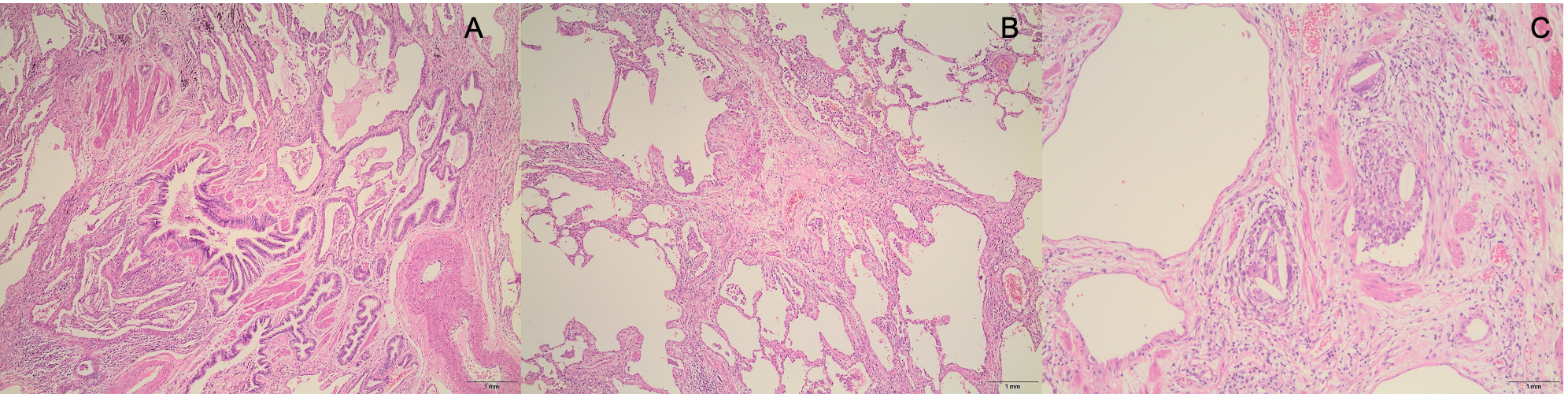


Supplementary Figure S4. Idiopathic pulmonary fibrosis with probable usual interstitial pneumonia (UIP) pattern on high resolution computed tomography and histological UIP pattern. A and B: Subpleural fibrosis and honeycombing (HE, 2,5x and 4x, respectively); C: Fibroblastic foci (HE, 20x).


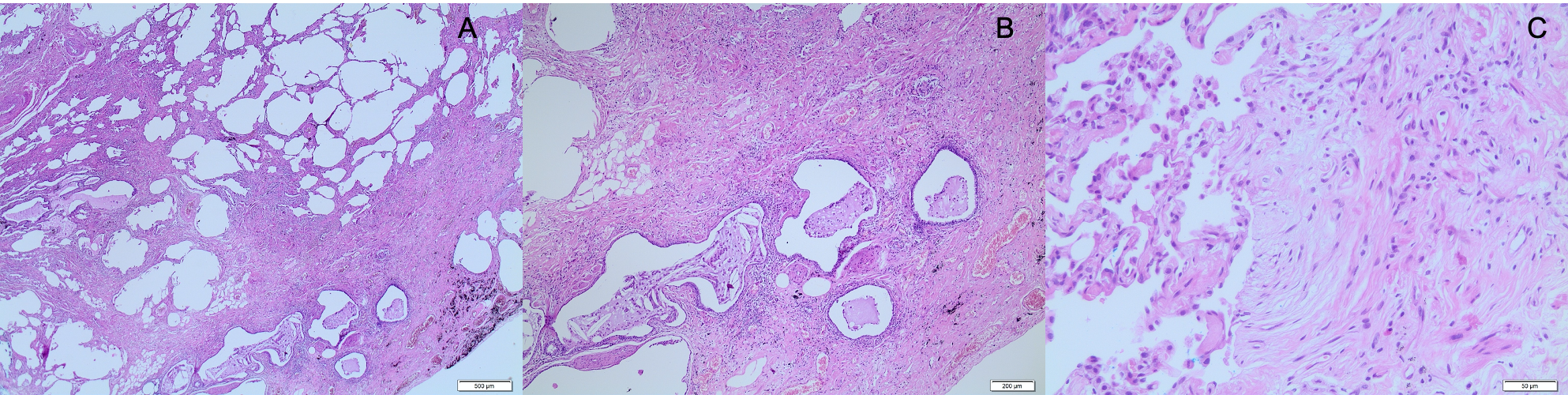


Supplementary Figure S5. Interstitial lung disease ascribed to gastroesophageal reflux disease with a probable usual interstitial pneumonia pattern on high resolution computed tomography and histological bronchiolocentric fibrosis (BF) pattern. A: Bronchiolocentric fibrosis (HE, 4x); B: Peribronchiolar metaplasia (HE, 4x); C: Focal honeycombing (HE, 4x).


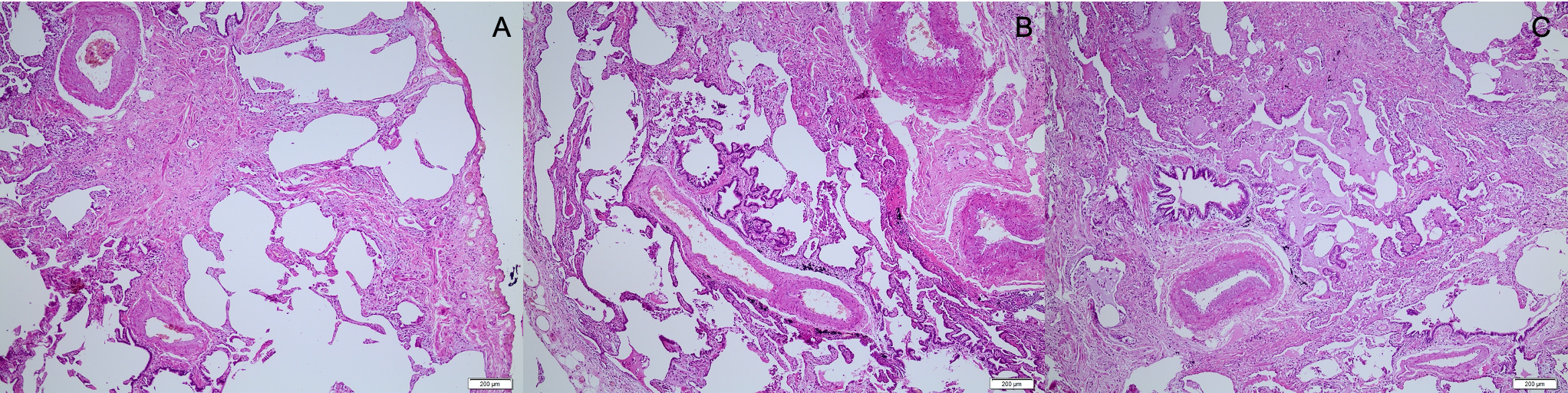


Supplementary Figure S6. Survival in the patients with probable usual interstitial pneumonia on high resolution computed tomography according to the diagnostic groups.


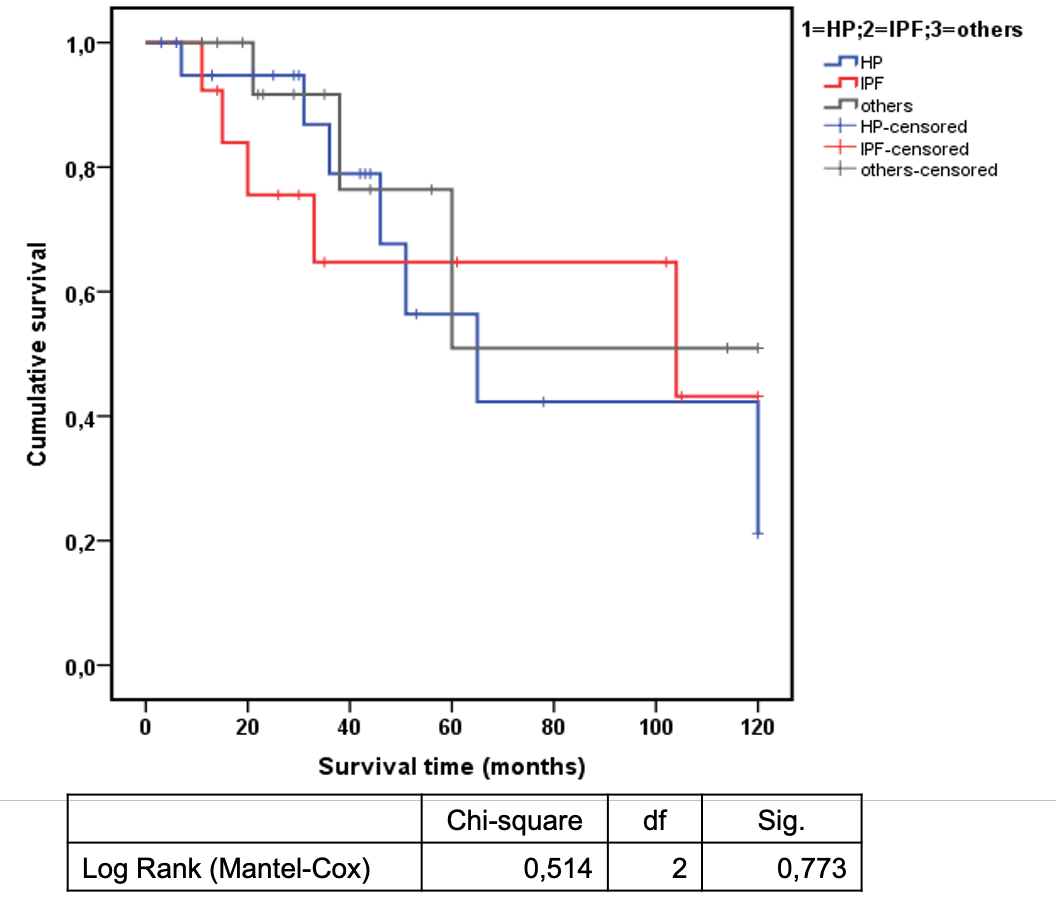


Supplementary Table S1. Clinical characteristics, functional characteristics, and HRCT findings of 50 patients with fibrotic hypersensitivity pneumonia, idiopathic pulmonary fibrosis, and other diagnoses

| Characteristic | | | | |
| --- | --- | --- | --- | --- |
|  | FHP  (n= 21*) | IPF  (n= 12) | Other  (n= 17) | p |
| Age in years 𝒙̅ ± SD | 65.2 ± 8.1 | 67.3 ± 6.5 | 63.6 ± 6.6 | 0.14 |
| Male, n (%) | 12 (57.1) | 9 (75.0) | 11 (64.7) | 0.60 |
| Presence of dyspnoea, n (%) | 18 (85.7) | 9 (75.0) | 14 (82.3) | 0.68 |
| Presence of cough, n (%) (n= 49) | 16 (76.2) | 8 (66.7) | 8 (50.0) | 0.24 |
| Duration of symptoms in months, median (Q1-Q3) (n= 47) | 24 (9-42) | 9 (3-12) | 24 (8-60) | 0.05 |
| Smoking history, n (%) (n= 47) | 9 (47.4) | 9 (75.0) | 8 (50.0) | 0.67 |
| Presence of environmental exposure, n (%) | 20 (95.2) | 2 (16.7) | 11 (64.7) | 0.001 |
| Symptoms of GERD, n (%) (n= 49) | 8 (40.0) | 3 (25.0) | 13 (68.4) | 0.24 |
| GERD confirmed, n (%) (n= 49) | 3 (15.0) | 2 (16.7) | 11 (64.7) | 0.002 |
| Familial history of ILD, n (%) | 0 | 0 | 5 (29.4) | 0.003 |
| Velcro crackles (n=48) | 14 (70.0) | 9 (75.0) | 11 (68.8) | 1.00 |
| FVC% predicted, 𝒙̅ ± SD | 82.2 ± 18.2 | 84.5 ± 15.3 | 74.6 ± 13.3 | 0.21 |
| SpO_2_ rest, 𝒙̅ ± SD (n= 44) | 94.7 ± 2.0 | 95.9 ± 1.8 | 94.8 ± 1.7 | 0.18 |
| SpO_2_ exercise, 𝒙̅ ± SD (n= 39) | 87.9 ± 5.3 | 91.5 ± 3.1 | 87.6 ± 5.8 | 0.20 |
| Honeycombing on HRCT**, n (%) | 4 (19.0) | 1 (8.3) | 3 (17.6) | 0.79 |

*In this group are included two patients with fibrotic hypersensitivity pneumonitis and ILD ascribed to GERD.** Cases with a single layer of honeycombing cysts located outside of the lower lobes. FHP: Fibrotic hypersensitivity pneumonitis; IPF: Idiopathic pulmonary fibrosis; GERD: Gastroesophageal reflux disease; FVC: Forced vital capacity; SpO_2_: Peripheral oxygen saturation; HRCT: High-resolution computed tomography.
